# Clinical evolutional aspects of chronic subdural haematomas – literature review

**Published:** 2015

**Authors:** IA Iliescu, AI Constantinescu

**Affiliations:** * Department of Neurosurgery, University Hospital Bucharest, Romania

**Keywords:** chronic subdural haematoma, antiplatelet drug, clinical manifestations

## Abstract

Apparently trivial, one of the most frequent pathologies in neurosurgical practice, chronic subdural haematoma, continues to be a challenge for the neurosurgeons both from the therapeutic and postoperatory complications point of view, taking into account that it is frequently met in elders, who usually present a complex pathology. The fact that, by definition, there is a latent period between the moment the brain injury, usually minor, occurs and the appearance of clinical symptomatology, frequently makes the trauma be ignored, this complicating the diagnosis and most of the times delaying the application of the adequate treatment.

Developing slowly in time, in weeks or months, the aspect that chronic subdural haematoma usually occurs in elders should not be neglected, its clinical symptomatology often debuting with memory and attention disorders, so that the patient is usually referred to psychiatrists or neurologists, only a paraclinical investigation (CT scan or MRI) being able to establish the diagnosis.

Even the appearance of the lateral signs is subjected to many diagnosis confusions because patients deny the existence of a trauma in over 50% of the cases.

**Abbreviations:** CT = computed tomography, MRI = magnetic resonance imaging, CSDH = chronic subdural haematoma, HMW = high molecular weight, F = frontal, T = temporal, P = parietal

## Introduction

Knowing the manifestation forms of chronic subdural haematomas is a first essential step in their diagnosis. Mostly affecting old people, the clinical manifestations of CSDH are often confused with the ones of other pathologies that are frequently present in elders. Being an extracerebral blood collection, which raises its dimensions in time and due to the fact that brain injury is frequently ignored, the symptomatology is often insidious, leading to errors in the diagnoses. Mainly, chronic subdural haematoma clinically manifests with psychic disorders in the initial phases, intracranial hypertension, and focal neurological signs.

**Clinical and physiopathological aspects**

After a minor traumatic brain injury, many times even without the loss of consciousness, there is usually an asymptomatic or oligosymptomatic (intermittent headaches, dizziness, occasional vomiting, memory and attention disorders) period, called “free interval”, with a duration of 4-12 weeks. According to McKissock et al. (1960), this free interval should have at least 3 weeks in order for the capsula that defines the physical aspect of the haematoma to be produced. Subsequently, focal signs represented by initial faciobrachial motor deficit, sometimes transitory, can appear, and the syndrome of intracranial hypertension begins to manifest itself completely through headaches, vomiting, and papilledema. The subsequent evolution is similar to all the intracranial expansive processes, the coma stage could appear if the initial signs are ignored or insufficiently investigated. 

From the clinical point of view, there are 3 distinct periods: 

1.first period – which corresponds to the traumatic brain injury 

2.second period – which coincides with the development of the chronic subdural haematoma and which usually manifests through the “free interval” previously presented 

3.third period – corresponding to the cerebrovascular decompensation, in which factors that favor the release of symptomatology interfere, leading to the break of the unstable balance between the subdural haematoma and the intracranial content or the raise of the volume of the haematoma over the compensation capacity of the intracranial content [**1**-**26**].

What should be mentioned is that, in the evolution of chronic subdural haematoma, sudden exacerbations can interfere, including the quick installation of the coma if a new bleeding occurs in the haematoma. This can happen due to a new trauma determined by the symptomatology of the initial chronic subdural haematoma (balance disorders, parethic motor deficit which can determine falls from the same level), or due to coagulopathy which many old people present as a result of anticoagulant or antiplatelets treatment, or diseases associated with liver failure, kidney failure, etc.

Most of the studies undergone evidence that in over 50% of the cases (between 52 and 58%), the alteration of the psychic functions, such as the confusion condition, memory disorders, are the first manifestations of a chronic subdural haematoma. 

Accordingly, most of the old patients who have been subsequently diagnosed with chronic subdural haematoma, initially present themselves to the neurologist or psychiatrist. What is worth mentioning is that most of the studies show a significant improvement and even the reversibility of the psychic disorders after an adequate treatment of CSDH. The younger persons and the ones who did not experience psychic disorders prior to the appearance of the haematoma, presented a more obvious reversibility and even a total reversibility of the psychic phenomena. 

Subsequently, headaches and focal neurological signs, such as hemiparesis can appear. Hemiparesis can initially be transitory and then constant. The focal epilepsy crises or generalized crises have also been mentioned in a low percent of the cases. Chronic subdural haematomas have frequently been initially labeled as strokes and only a CT scan or a MRI examination could offer a clear diagnosis. 

A study done on 500 patients with symptomatic subdural haematomas evidenced that over a half of the patients (63%) present to the physician with only one symptom, as for example walking and balance disorders, hemiparesis, headache, psychic disorders, rarely urinary incontinence, alterations of the consciousness, etc., and 48% of the patients present to the examination with an association of 2 clinical signs, such as walking disorders and hemiparesis, walking and psychic disorders, headache and hemiparesis or walking disorders and urinary incontinence. Only 9% of the patients presented more than 2 symptoms or clinical signs [**27**-**32**].

Regarding the way a chronic subdural haematoma expands; various studies were undergone trying to explain its expansion in time. None of these studies managed to totally elucidate the way a chronic subdural haematoma expands, which indicates that this phenomena depends on many factors and numerous processes are involved in the process. As for the history of this issue, Putnam and Cushing (1925) have launched the hypothesis of repeated bleedings at the capsula level and Gardner (1932) and then Weir (1971) have launched the hypothesis of the osmotic oncotic mechanism. 

A study undergone by Japanese researchers has analyzed the role of the endothelial vascular growth factor and the fibroblastic growth factor in chronic subdural haematomas. The endothelial vascular growth factor in vitro has an extremely high mitogenic action on the endothelial cells and, in vivo, it induces angiogenesis. 

The conclusion of the study was that in the case of the haematoma, the concentration of the endothelial vascular growth factor is on average 28 times higher than in the serum. The concentration of the fibroblastic growth factor in the haematoma had similar values with the one in the serum or even lower, which means that there is no correlation between the fibroblastic growth factor and the endothelial vascular growth factor. Taking into account that this endothelial growth factor has a role only in stimulating the growth of the endothelial cells, and it is a very powerful plasminogen activator, it is considered an important autocrine growth factor of these subdural haematomas and that it induces neovascularization, excessive vascular permeability and rebleeding, having an important role in the persistency and expansion of chronic subdural haematomas [**18**]. 

Other Japanese researchers have studied the kinin-kallikrein system role in the vascular permeability and the regulation of fibrinolysis and coagulation in chronic subdural haematomas. Bradikinin, the final product of the kinin-kallikrein system is a powerful mediator that raises the vascular permeability. Its concentration in haematomas was significantly higher than in the skin. In the same time, the activity of the prekallikrein and of HMW-kininogen was significantly more reduced than the one in the skin of the patients. 

These demonstrate that the activation of kinin-kallikrein system in the case of chronic subdural haematomas. While determining the fibrinogen concentration and the degrading products of fibrinogen in the haematoma liquid, it was noticed that the fibrinogen exists in low amounts while fibrin and the degrading products of fibrinogen exist in high amounts as compared to the skin. A high concentration was also found in the case of plasminogen activator both in the haematoma and in its parietal membrane. These determinations lead to the idea that a local hyperfibrinolysis takes place both in the haematoma and in its parietal membrane and the hyperfibrinolysis would be the determinant factor of chronic subdural haematomas extension. Moreover, hyperfibrinolysis leads to repetitive micro bleedings from the parietal membrane in the cavity of the haematoma, determining its expansion. The kinin-kallikrein system is made up of prekallikrein, HMW-kininogen (a kininogen with a great molecular weight) and bradikinin. Prekallikrein is the inactive predecessor of kallikrein and it is located in the skin. The skin is activated and transformed into kallikrein by Hageman XII factor. As far as kallikrein is concerned, it transforms the HMW-kininogen in bradikinin. The lowering of the prekallikrein and HMW-kininogen concentration showed that the kinin-kallikrein system was activated. 

In the study of Japanese researchers, the plasmatic values of pre-kallikrein and of HMW – kininogen are normal, while at the level of the haematoma these have been significantly high, which indicates a local activation of this system at the level of the parietal membrane of the haematoma [**17**]. 

The liquid in the haematoma contains both leukocytes and erythrocytes marked with Crom-51. Ito has demonstrated that there are daily micro hemorrhages from its parietal membrane in the cavity of the haematoma. Moreover, a growth of the eosinophils both in the membrane and in the cavity of the haematoma was demonstrated. The obvious conclusion is that the sanguine cells pass from the capillaries in the interstitial tissue of the parietal membrane and from this place, they reach the cavity of the haematoma. This is due to a growth of the vascular permeability. Many factors such as bradikinin, histamine, serotonin and prostaglandins have been demonstrated to take part in the growth of the vascular permeability. At the level of the parietal membrane of the chronic subdural haematoma, which in light of the microscopic studies and the enzymatic determinations, is looked at as an inflammatory factor by some researchers and as a repairing factor by other researchers, all these factors can be activated. Bradikinin is known as one of the most important mediators of the growth of vascular permeability, having an almost 20 times higher effect than histamine and prostaglandins. As a result, this study sustains the idea that bradikinin generated by the activation of kinin-kallikrein system is the main factor responsible for the growth of vascular permeability in cases of chronic subdural haematomas [**22**]. 

To conclude with, most of the studies undergone until recently, have concentrated on the parietal membrane of the chronic subdural haematoma, both as a microscopic structure and as an enzymatic activity, in which case various substances biologically active were found. 

Beside the endothelial vascular growth factor previously described, the plasminogen tissue activator was also identified, inflammatory cytokines of interlukin-6 and interleukin-8 type and E2 prostaglandin, this being the reason why the parietal membrane was considered an inflammatory phenomena by some researchers. In addition, the plasminogen tissue activator has value of 3 times higher in the parietal membrane than in the dura mater, while the visceral membrane of the haematoma does not contain this factor, which exudates from the extremely vascularized parietal membrane, transforms plasminogen in plasmine in the subdural haematoma, by neutralizing the fibrinogen and fibrin and favoring continuous micro bleedings. On the other side, the antiplasmin in the haematoma is less concentrated than in the venous blood. Moreover, it was demonstrated that there is a linear evolution between the E2 prostaglandin concentration in the liquid of the haematoma and its age, which led to the conclusion that cyclooxygenase-2 (the enzyme involved in E2 prostaglandin synthesis) plays a very important role in the development of chronic subdural haematoma. 

It is important to underline that a study done by some Canadian researchers, regarding the osmolarity of the liquid in chronic subdural haematoma compared with the one of the venous blood and the cephalorachidian fluid, has shown that there are no significant osmolarity differences between these fluids. It is considered that the late appearance of the symptoms is due to the constant micro bleedings in the parietal membrane or/ and effusion through the microcapillaries of albumin and plasma because this study does not support the concept according to which the raise in dimensions of the haematoma is the result of an osmotic mechanism [**21**].

Generally, the clinical picture is considered to be represented by the often minor craniocerebral injury, the free interval of 3-12 weeks (during this interval headaches and dizziness can appear) and then the gradual installation of neurological focal signs (pareses, speech disorders, etc.) and finally the intracranial hypertension signs. 

**Fig. 1:**
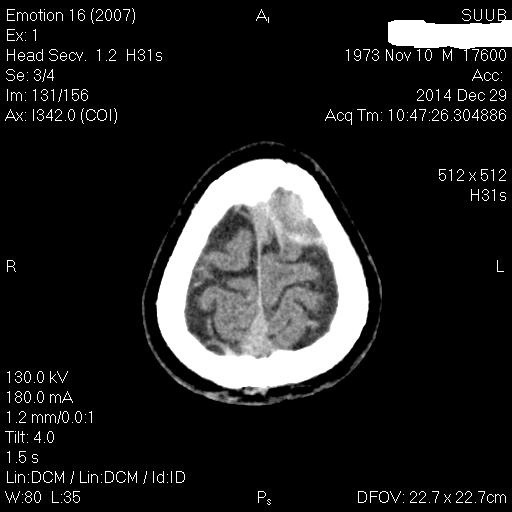
Axial CT scan which evidences a left frontal serohematic effusion in patient M.F. of 41 years old

**Fig. 2:**
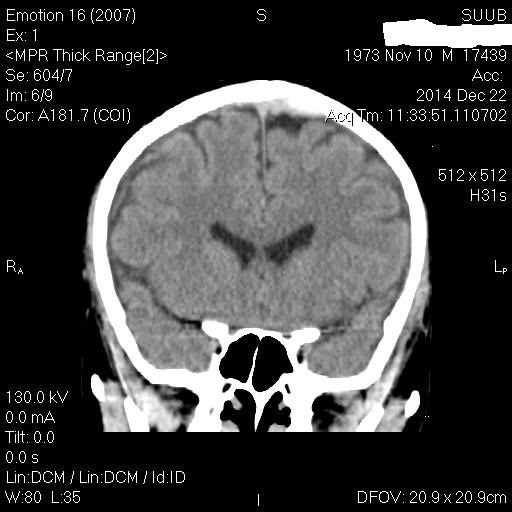
CT scan (coronal plane reconstruction) which evidences the high left frontal serohematic effusion

**Fig. 3:**
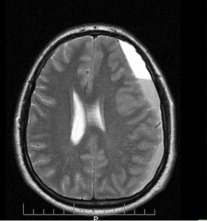
MRI aspect with left brain hemisphere subdural haematoma in patient M.F. of 41 years old (45 days after the initial discharge from hospital)

**Fig. 4:**
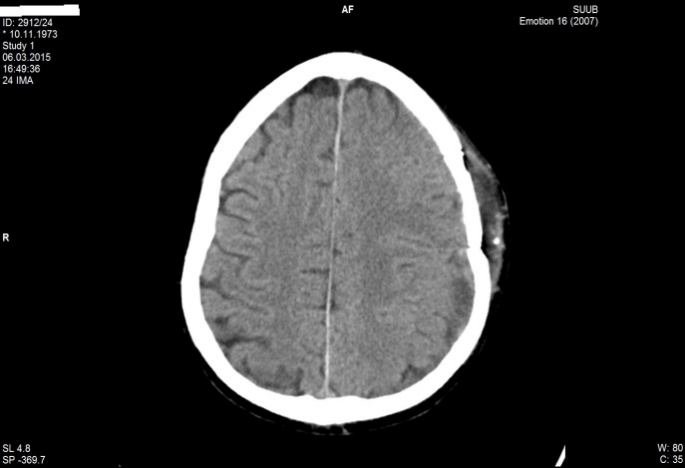
Postoperatory CT aspect in patient M.F. of 41 years old

**Presentation of clinical cases.** 3 clinical cases with different evolution aspects will be presented: 

**Case 1**. The first case was of a 41-year-old man, M.F., without a significant pathological history, who presented to the Emergency Room with intense headache and dizziness installed after an aggression craniocerebral injury. The objective neurological examination did not show neurological deficits, the patient being conscious, cooperative, GCS-15. The cerebral CT examination showed a left linear paramedian F-P cranial fracture, without post-traumatic intracranial injuries. The patient was hospitalized for observation. A CT examination was done at 4 days; the patient’s state clinically improved without neurological deficits and was proposed for discharge, and showed a left parasagittal F-P extracerebral hematic line, with a thickness of 6 mm at the level of the vertex (**Fig. 1**, **2**). Another CT examination was done at 10 days, the patient also presenting no neurological deficits, no other pains; the GCS-15 evidenced the resorption of the hematic line with the left soft F-P hygroma line, the patient finally being discharged. In 45 days from the discharge, the patient presented in the clinic accusing an intense accentuated headache for almost 2 weeks, which could not be treated while being administered usual antalgics. The MRI examination showed a left chronic F-T-P subdural haematoma, with a thickness of almost 18 mm (**Fig. 3**), for which a surgery through the volet was performed. After the removal of the haematoma, a drainage system was installed and was removed after 48 hours. The control brain CT was performed 7 days postoperatory and highlighted a residual serohematic line (**Fig. 4**) which was not visualized in the control MRI that was performed 45 days postoperatory. 

This case was presented because it had a two stages evolution, in a young patient, without a pathological history. 

**Case 2**. The patient was a 71-year-old man from rural area, suffering from diabetes, while being administered oral antidiabetic medicines, presenting sequelae after a septal myocardial infarction and who was following a chronic treatment with platelets antiaggregants (Aspenter), presented to the Emergency Room for headaches, walking and balance disorders, memory disorders, muscle weakness in the left limbs, a symptomatology which debuted 1 week before and which accentuated progressively. The family of the patient accused a minor craniocerebral injury produced by an accidental hit on the doorframe, 6 weeks before the hospitalization. Moreover, the family of the patient mentioned a crisis with a loss of consciousness 24 hours before the admission into hospital. 

**Fig. 5:**
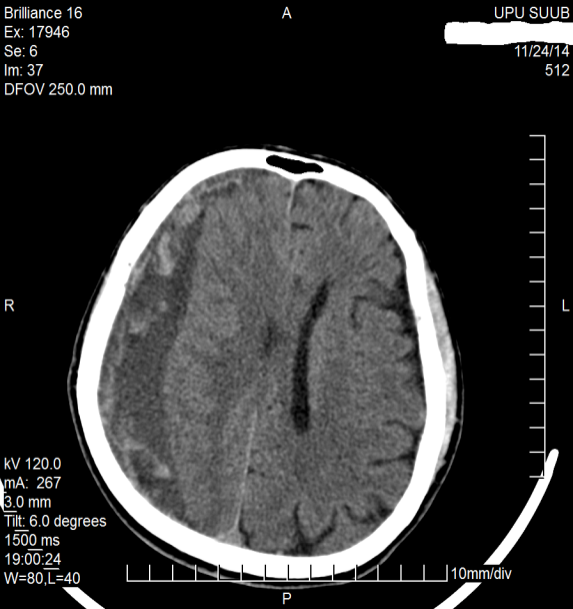
Postoperative CT aspect in patient B.I. of 71 years old; chronic subdural haematoma with a light recent bleeding of right brain hemisphere

The objective neurological examination evidenced a motor deficit of the left side limbs, walking and balance disorders, confusion state. The brain CT examination evidenced a chronic subdural haematoma of the right brain hemisphere, with a thickness of 27 mm, a light recent bleeding and a mass effect on the ventricular system, which was moved to the left (**Fig. 5**). 

After the correction of the glucose level (the initial value of 367mg/ 100ml) by insulin administration according to the nutritionist’s recommendations and the stopping of the platelet antiaggregant treatment, surgery was undergone 48 hours from the admission and the chronic subdural haematoma of right brain hemisphere was evacuated through a right fronto-temporo-parietal volet. The postoperatory evolution was favorable with the improvement of the motor deficit of the left side limbs, the improvement of the confusion state and the walking and balance disorders. Moreover, during the admission period, the patient did not present crises of loss of consciousness. What should also be mentioned is that an anticonvulsive treatment was applied from the first day of hospitalization. 

**Fig. 6:**
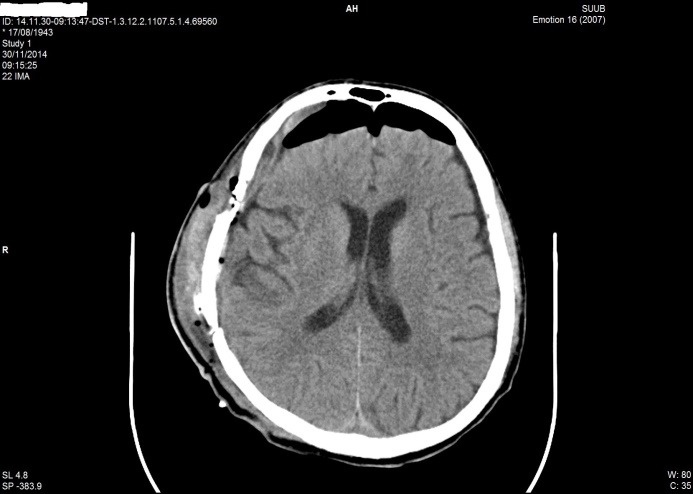
Postoperative CT aspect in patient B.I. of 71 years old; evacuated subdural haematoma, re-expansion brain, ventricular system on the median line

The cardiologic consultation recommended the treatment repeating with Aspenter after solving the neurosurgical problem, after performing the brain CT control, 7 days postoperatory, the patient was discharged with an anticonvulsive treatment, oral antidiabetic medication and a recommendation to retake the treatment with Aspenter after another week (**Fig. 6**). 

The case is interesting because it was met in a patient with minor craniocerebral injury while under antiaggregant platelet treatment with Aspenter, presenting an evolution of the haematoma of about 6 weeks and who, 24 hours before hospital admission, manifested a loss of consciousness crisis, possibly due to a recent minor bleeding. What should also be mentioned is that the patient continued his antiaggregant treatment 1 week after discharge, according to the indications of the cardiologist and neurologist.

**Case 3**. The 3rd case is of a patient of 87 years old, P.M., without significant pathological history, who did not follow any chronic treatment. Due to a minor craniocerebral injury (house accident – an accidental fall from a chair) presented to the Emergency Room with headache and dizziness. 

**Fig. 7:**
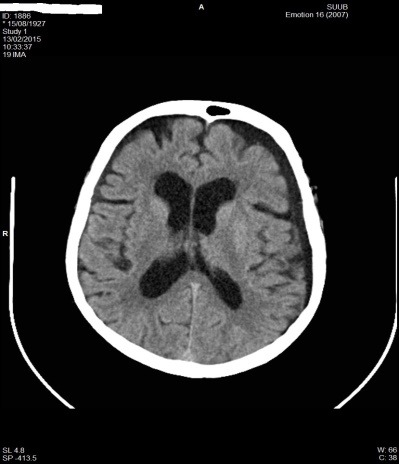
CT aspect with a left F-T hygroma in patient P.M. of 87 years old

**Fig. 8:**
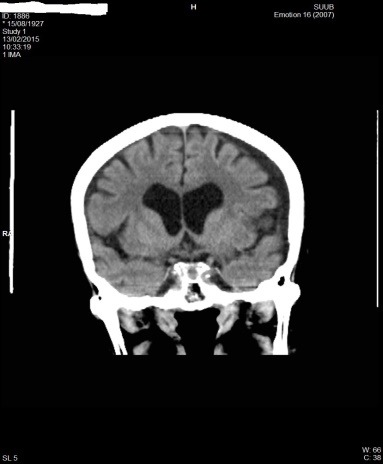
CT aspect (coronal plane) with left F-T hygroma in patient P.M. of 87 years old

**Fig. 9:**
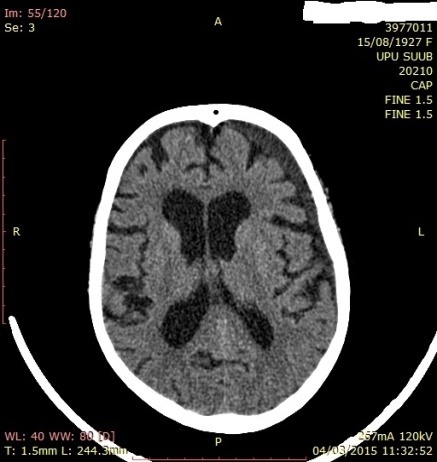
CT aspect with left F-T hygroma in patient P.M. of 87 years old on discharge and under neurosurgical surveillance

The objective neurological examination did not show neurological deficits, the patient was conscious, cooperating GCS-15. The brain CT examination evidenced a light interhemispheric and periportal hematic line. The patient was hospitalized for surveillance, the CT examination was repeated at 7 days evidencing the appearance of a left F-T hygroma and the resorption of the initial interhemispheric and periportal hematic densities (**Fig. 7**,**8**). Another CT examination repeated at 21 days evidenced the significant resorption of left F-T-P hygroma, the patient being discharged while being conscious, cooperating, without neurological deficits, without accusing pain, GCS-15, with the recommendation of neurosurgical reevaluation (**Fig. 9**). 

**Fig. 10:**
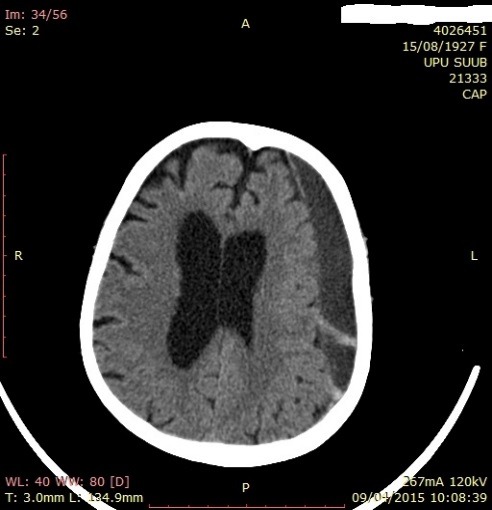
CT scan at readmission after 30 days in patient P.M. of 87 years old; left brain hemisphere chronic subdural haematoma

**Fig. 11:**
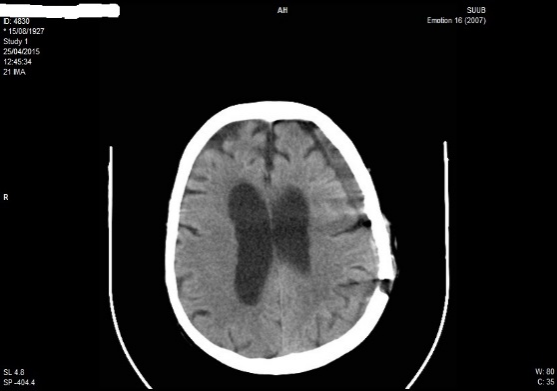
CT scan at 5 days postoperatory in patient P.M. of 87 years old; a little left F-T restant serohematic line can be observed, without a compressive effect on the brain

At 30 days from the discharge (almost 2 months from the initial trauma), the patient reintegrated for a clinical-imagistic reevaluation. The objective neurological examination did not detect focal neurological deficits but only a light confusion state. The CT examination of the brain evidenced a left-brain hemisphere chronic subdural haematoma, with rare instances of hematic density at the level of the haematoma, a thickness of 20mm, which determined a soft mass effect to the ventricular system, which appeared as shifted to the right with 6mm (**Fig. 10**). After the cardiologic examination which detected a left bundle branch block (LBBB) without any treatment indication and a light hypertension, the echocardiographic examination according to the age pattern, surgery was performed and the chronic subdural haematoma was evacuated through the left burr-hole. 

3 hours postoperatory, the patient was conscious, cooperative, did not present neurological deficits; at 24 hours postoperatory she became agitated from the psycho-motor point of view, confused, has periods of drowsiness, without focal neurological deficits; the CT brain examination at 24 hours postoperatory evidenced a little remnant of left frontal sero-hematic trace, without a compressive effect on the subajacent brain and without a mass effect on the ventricular system. Subsequently, the neurological state of the patient depreciated in the sense that she presented acute drowsiness and, at nociceptive stimulation mobilized the limbs equally bilaterally, her tracheobronchial tree was loaded. A CT of the thorax was undergone and a brain CT was repeated at 5 days postoperatively (**Fig. 11**). The thorax examination did not show pulmonary pathological aspects and the brain examination evidenced the reduction in thickness of the remnant sero-hematic trace. An aggressive treatment based on antibiotics was started, tapping and turning from side to the other maneuvers were constantly performed in order to avoid the appearance of trophic injuries. Despite the aggressive treatment with antibiotics, the nursing maneuvers, the follow-up of the biological constants, the patients’ condition progressively depreciated and, at 10 days postoperatory, she suffered a cardiopulmonary arrest and exitus, from which she could not be revived. 

We have presented this case to underline the aspect that, even it seems a simple affection, due to the fact that it predominantly appears in elders, chronic subdural haematoma can raise major postoperatory problems, sometimes with a bad prognosis. These are due to the vascular fragility of the old person’s brain, the diminished general resistance of the old person’s organism, the unavoidable deposit of the atheromatous plaques at the vascular levels with the already known consequences, the degenerative modifications of the cervical spondyloarthritis disk which limit the mobility of the head and maybe can determine the compression on the vertebral arteries in cases of surgeries of the brain, complex pathologies associated to the elders. 

## Discussions, conclusions 

Chronic subdural haematomas are a neurosurgical pathology constantly growing, especially in the conditions of CT scan examination possibility. The incidence of these haematomas is of 1,7-18/ 100.000 inhabitants in the group of patients over 65 years old. The medium age of the patients with chronic subdural haematoma is 63 years and, taking into account the aging rhythm of the population in the next 15 years, the incidence is expected to double. Moreover, chronic subdural haematomas represent an important cause of dementia and neurological deficits in elders but, fortunately, reversible. The growth in the clinical susceptibility and the access to paraclinical investigations allow a precocious treatment, which sometimes removes the risk of an unfavorable prognosis. 

What should also be mentioned is the INRO (International Neurotrauma Research Organization) study which evidences significant differences between the mortality rate in the countries with a higher GDP/ inhabitant and the ones with a lower GDP/ inhabitant (respectively amounts of money allocated per patient). The major problems are generated not by the lack of therapeutical protocols (the participant countries had the same protocols) but by allocation of the therapeutical resources, the countries with a lower GDP/ inhabitant being practically in the impossibility of putting into practice those protocols. The road accident remains the main cause of TCC in all the industrialized countries. The second cause in TCC etiology is represented by the falls from the same level or from a certain height. The data of a preliminary investigation in Romania, undergone in 1997 by the Neurotrauma Group of the Romanian Society of Neurosurgery have evidenced a TCC share of 25-95% in the neurosurgery departments in Romania, with a mortality rate in severe TCC of 60-90%, while the same indicator was of 31% in 1996, in the countries of the European Community. 

Accordingly, taking into account the fact that most of the statistics evidence a growth of the elder population with all the pathological issues associated to this category of age, a growth of the incidence of chronic subdural haematomas in the specialty services has occurred, the problem of chronic subdural haematoma being important and needing appropriation by all the neurosurgeons as far as the surgical treatment and the probable postoperatory evolution are concerned. 

As a personal observation, if after a neurosurgical intervention at the brain level, no matter how simple it may apparently seem, an old person is not precociously mobilized, the risks of developing postoperative complications exponentially raise and this leads to a growth of the hospitalization period, a raise of the costs of medical services and, finally, yet importantly, it can affect the life prognosis of the patient. 
